# A novel functional cross-interaction between opioid and pheromone signaling may be involved in stress avoidance in *Caenorhabditis elegans*

**DOI:** 10.1038/s41598-020-64567-3

**Published:** 2020-05-05

**Authors:** Jun Young Park, Mi Cheong Cheong, Jin-Young Cho, Hyeon-Sook Koo, Young-Ki Paik

**Affiliations:** 1 Interdisciplinary Program in Integrative Omics for Biomedical Science, Yonsei University, Seoul, 03722 Korea; 2 Yonsei Proteome Research Center, Yonsei University, Seoul, 03722 Korea; 3 Department of Pharmacology, UT Southwestern Medical Center at Dallas, Dallas, TX 75390 USA; 4 Department of Biochemistry, College of Life Science and Biotechnology, Yonsei University, Seoul, 03722 Korea

**Keywords:** Developmental biology, Caenorhabditis elegans

## Abstract

Upon sensing starvation stress, *Caenorhabditis elegans* larvae (L2d) elicit two seemingly opposing behaviors to escape from the stressful condition: food-seeking roaming mediated by the opioid peptide NLP-24 and dauer formation mediated by pheromones. Because opioid and pheromone signals both originate in ASI chemosensory neurons, we hypothesized that they might act sequentially or competitively to avoid starvation stress. Our data shows that NPR-17 opioid receptor signaling suppressed pheromone biosynthesis and the overexpression of opioid genes disturbed dauer formation. Likewise, DAF-37 pheromone receptor signaling negatively modulated *nlp-24* expression in the ASI neurons. Under short-term starvation (STS, 3 h), both pheromone and opioid signaling were downregulated in *gpa-3* mutants. Surprisingly, the *gpa-3;nlp-24* double mutants exhibited much higher dauer formation than seen in either of the single mutants. Under long-term starvation (LTS, >24 h), the stress-activated SKN-1a downregulated opioid signaling and then enhanced dauer formation. Both insulin and serotonin stimulated opioid signaling, whereas NHR-69 suppressed opioid signaling. Thus, GPA-3 and SKN-1a are proposed to regulate cross-antagonistic interaction between opioids and pheromones in a cell-specific manner. These regulatory functions are suggested to be exerted via the selective interaction of GPA-3 with NPR-17 and site-specific SKN-1 binding to the promoter of *nlp-24* to facilitate stress avoidance.

## Introduction

Animals, including C*aenorhabditis elegans*, are confronted with a variety of environmental stresses such as starvation, temperature changes, and increased population density, among others. In some cases, the best strategy for handling such insults might be to avoid them. Regardless of its duration, stress generally perturbs homeostasis and elicits responses that lead to critical cellular processes^1^, evolutionarily-conserved behavioral, endocrinal, and cognitive outcomes to ensure survival^2^. When early-stage *C. elegans* larvae encounter starvation stress, they often elicit two seemingly opposing behavioral responses: *(i)* their pharyngeal pumping rate is stimulated to drive foraging^3^ or *(ii)* they enter the dauer stage, an alternative third larval stage. The foraging behavioral response is governed by endogenous opioid signaling^4^. Additionally, two groups of chemosensory and mechanosensory glutamatergic neurons are shown to trigger food-seeking behaviors^5^. The dauer formation is mainly induced by pheromones through the modulatory actions of insulin and TGF-β signaling^6–10^.

The *C. elegans* endogenous opioid signaling system consists of opioid ligands (e.g., NLP-24) and their receptors (e.g., NPR-17, a homolog of mammalian opioid receptors)^4^. In mammals, endogenous (e.g., endorphins) or synthetic (e.g., fentanyl) opioids have been implicated in stress avoidance by either attenuating stress responses or by dulling stress-induced pain^11–14^. The pheromone signaling system consists of ascaroside pheromones (e.g., daumone, ascr#2, 3)^15,16^ and at least five molecular components, although others likely exist^17,18^. The molecular components are pheromone binding (DAF-37), signal sensing perception (GPA-3), neuronal transmission (EAT-4), glutamate processing (GLNA-3), and eliciting of the final behavioral outputs (e.g., the repulsive response) by gate molecules (e.g., MGL-1)^18,19^. Feeding via pharyngeal pumping ceases during when early *C. elegans* larvae enter the dauer stage, which is mainly mediated by pheromones^3,7,10,15,20^. This interconnection between two behaviors (i.e., the cessation of feeding and entry into dauer) suggests that opioid and pheromone signaling pathways might initiate sequentially in the ASI neurons and mutually influence each other under starvation stress in *C. elegans*. Apparently, opioid and pheromone signaling seem to play the similar roles in a worm’s early survival strategy in response to starvation stress. However, the functional relationship of these two signaling pathways during stress avoidance and the mechanism by which pheromone-mediated dauer formation leads to the cessation of opioid-governed pharyngeal pumping are not completely understood.

**Figure 1:**
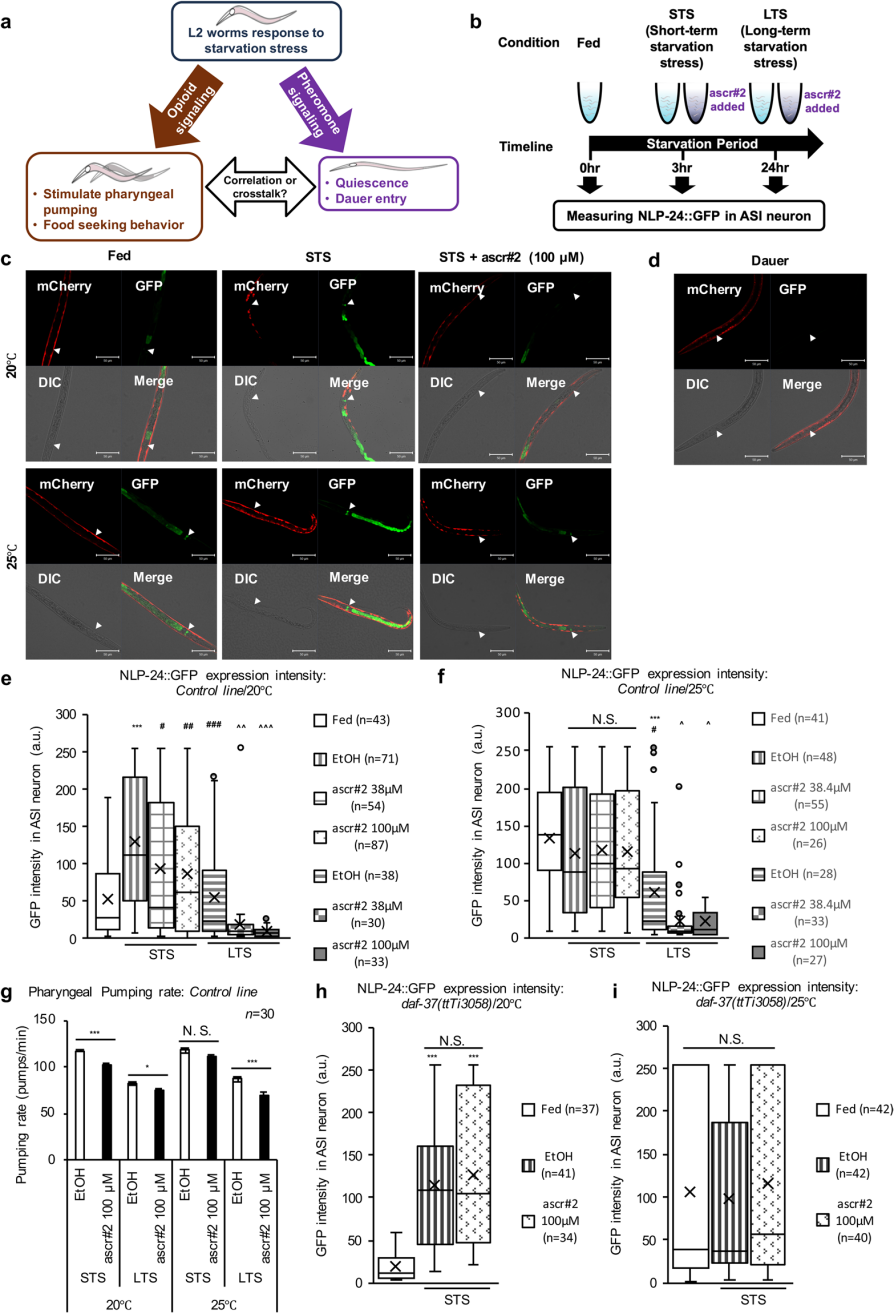
The effects of stress and ascaroside pheromone on *nlp-24* expression. (**a**) Relationship between opioid and pheromone signaling pathways for stress avoidance. **(b)** Experimental design of measuring NLP-24::GFP under starvation stress. **(c,d)** NLP-24::GFP was expressed in ASI neurons under fed, starved, and starved with ascr#2 conditions (**c)** at 20°C, 25°C, and **(d)** at dauer state. For each condition, the images were taken using a Zeiss LSM880 confocal microscope at 40x magnification with a water-immersion lens. A white arrow indicates the position of the ASI neuron. Bar 50 μm. **(e,f,h,i)** The expression of NLP-24::GFP in the *control line* (**e)** at 20°C, **(f)** at 25°C, in the *daf-37(ttTi3058)* background **(h)** at 20°C, and **(i)** at 25°C. Each box plot was generated by using ImageJ to analyze the GFP fluorescence intensities in the ASI neurons in the 8-bit images taken under each condition (values from 0 ~ 255). Each measured value (in arbitrary units, a.u.) from the individual worms constituted a box plot specific for each condition. Each box in the box plot was represents the inter-quartile range, the bar in the box represents median, and the X symbols represents the mean of the measured values under each condition. *, **, and *** indicate *p-*values <0.05, <0.01, and <0.001, respectively, compared to the fed condition. #, ##, and ### indicate *p-*values <0.05, <0.01, and <0.001, respectively, compared to the STS condition. ^, ^^, and ^^^ indicate *p-*values <0.05, <0.01, and <0.001, respectively, compared to the LTS condition. N.S. indicates no statistically significant difference. **(g)** Pharyngeal pumping rates of *control line* (*nlp-24;*E*x[pnlp-24::nlp-24::*GF*P]*). Each condition was same as measuring NLP-24::GFP fluorescence experiment. *, **, and *** indicate *p-*values <0.05, <0.01, and <0.001, respectively. All *p-*values were calculated by unpaired *t-*test.

Because both signaling pathways originate in ASI neurons, which likely produce a coherent common readout upon sensing starvation, we hypothesized the pathways might interact functionally during starvation stress (Fig. 1a). To test this hypothesis, we sought to answer two sets of questions: (*i*) What is the nature of the interaction? Is it cooperative, competitive, or other? (*ii*) What factors might be integrated by these two signals to facilitate stress avoidance? In this work, we demonstrate a likely functional antagonistic relationship between the opioid and pheromone signaling pathways during different duration starvation stress in which GPA-3 and SKN-1 may play the key roles. We also show that these two pathways appear to be intertwined with several other cellular signaling pathways. Our results could support the use of *C. elegans* as a convenient model organism for studying the molecular processes underlying opioid metabolism in vertebrate.

## Results

### Pheromone signaling negatively modulated the opioid signaling in early starvation

To examine what type of functional interactions might exist between opioid and pheromone signaling, we first mined the expression data contained in dauerDB (www.dauerdb.org)^21^. We found reduced *nlp-24* transcription might be linked to dauer formation (Supplementary Fig. S1), suggesting a possibility of a negative relationship between them. To test this possibility, we assessed changes in the *nlp-24* expression under two stress conditions, short- (~3 hr; STS) and long-term starvation (>24 hr; LTS). We conducted these assessments in the presence or absence of ascr#2^22^, a DAF-37 ligand^19^, under optimal dauer assay conditions^15,23,24^ (Fig. 1b, Supplementary Fig. S2). As expected, the *nlp-24* expression in the ASI neurons in a transgenic rescue line (*nlp-24;Ex[pnlp-24::nlp-24::GFP]*, designated as the *control line*), was higher in stressed worms (e.g., starvation and temperature) (Fig. 1c). In agreement with the dauerDB data (Supplementary Fig. S1) and in contrast to the starvation results, the pheromone treatment decreased the *nlp-24* expression, and entry into the dauer stage abolished it entirely (Fig. 1d). The NLP-24 protein level in the ASI neurons decreased during STS at 20 °C upon the addition of pheromones compared to the same conditions at 25 °C (Fig. 1e,f). Note that under both starvation and temperature stress conditions, the addition of more pheromone did not seem to further suppress *nlp-24* expression (i.e., 38 μM vs.100 μM ascr#2) (Fig. 1e,f). This result suggests that even in the presence of pheromones, L2 worms do not usually enter dauer under fed condition in which *nlp-24* may autonomously act together to inhibit such pheromone action (Supplementary Fig. S3)^15^. Furthermore, *nlp-24* expression was reduced during long-term starvation without exogenous pheromone, whereas *nlp-24* expression was increased during short-term starvation (Fig. 1e,f). These effects are presumably due to the long-term stress experienced by the *control line* during which *nlp-24* expression may be reduced by endogenously synthesized pheromones in the body. Taken together, these findings suggest that pheromones exerted a negative effect on opioid signaling during starvation in ASI neurons.

To investigate whether the pheromone-dependent suppression in *nlp-24* expression also influenced feeding activity, we next measured the pharyngeal pumping rates under both starvation conditions, as previously described^4^. The pharyngeal pumping rate was reduced in the presence of pheromones at both temperatures (Fig. 1g), mirroring the *nlp-24* expression pattern (Fig. 1e,f). This finding suggests pheromone treatment resulted in a parallel decrease in both *nlp-24* expression and pharyngeal pumping rate.

Having confirmed the negative effect of the pheromone ligand on the *nlp-24* expression during STS, we also wanted to examine whether the DAF-37 pheromone receptor, like its ligand, exerted the same negative action against opioid expression. To test this, we measured the NLP-24 expression in the ASI neurons and the dauer formation ratio in the *daf-37(ttTi3058)* mutant background, which is known to be dauer-defective^19^. The DAF-37 pheromone receptor-mediated sensing signaling also negatively modulated opioid (*nlp-24*) expression; there was no clear difference in the *nlp-24* expression regardless of the presence of pheromones at both 20 and 25 °C (Fig. 1h,i). This finding suggests that the DAF-37 receptor-mediated pheromone signaling negatively regulate the expression of *nlp-24*.

### Pre-requisite for the negative modulatory effect of pheromones on the opioid signaling

**Figure 2:**
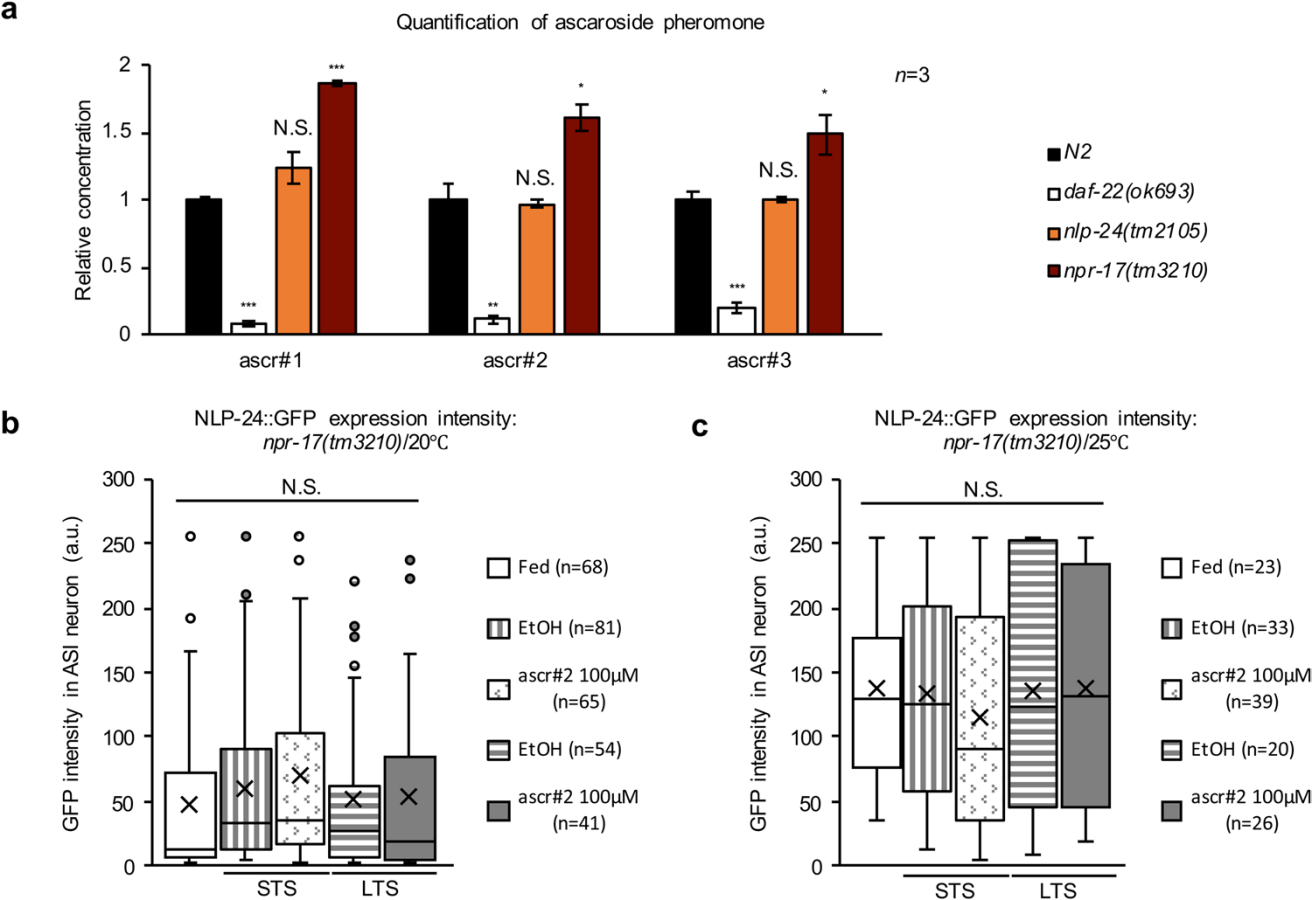
The role of opioid-like signaling in ascaroside pheromone-induced physiology. (**a**) The amount of ascaroside pheromone biosynthesis in the N2 wildtype, *nlp-24(tm2105)*, *npr-17(tm3210)*, and *daf-22(ok693)* as a negative control (*n* = 3). The data are represented as the mean with SEM from more than three different experiments with three technical repeats. *, **, and *** indicate *p-*values <0.05, <0.01, and <0.001, respectively. N.S. indicates no statistically significant difference. **(b,c)** The expression of NLP-24:**:**GFP in the ASI neurons under fed, STS, LTS, and with ascr#2 conditions in the *npr-17(tm3210)* background **(b)** at 20°C, **(c)** at 25°C. Method and data processing for measured values are the same as in the legend of Fig. 1. Each box in the box plot was represents the inter-quartile range, the bar in the box represents median, and the X symbols represents the mean of the measured values under each condition. N.S. indicates no statistically significant difference. All *p-*values were calculated by unpaired *t-*test.

Given the negative effect of pheromone signaling on opioid signaling (see Fig. 1e,f), we next questioned whether opioid signaling affected the production of pheromones. We measured the relative rate of pheromone biosynthesis in *nlp-24* or *npr-17* mutants which are defective in opioid signaling response, and the production of pheromones was increased in the *npr-17* mutant but not the *nlp-24* mutant. Compared to the wildtype, there was a 49–87% increase in the levels of three major ascaroside pheromones in the *npr-17* mutants: ascr#1 ($87 \% \pm 3$, *n* = 3), ascr#2 ($62 \% \pm 10$, *n* = 3), and ascr#3 ($49 \% \pm 15$, *n* = 3) (Fig. 2a). This finding suggests that the NPR-17 receptor-mediated signaling might negatively regulate pheromone biosynthesis, like the effect of pheromone receptor signaling against the expression of *nlp-24*. The reason behind this phenomenon remains unclear.

To investigate the molecular components of opioid regulation by pheromone signaling that are coupled to the pheromone biosynthesis^17,24,25^, we measured the *nlp-24* expression level in *daf-22(ok693)* mutants that were deficient in pheromone biosynthesis. Pheromones did not negatively affect the opioid ligand expression in this mutant; there were no substantial increases in the *nlp-24* expression under both STS and LTS compared to the *control line* at 20 °C (Supplementary Fig. S4a and Supplementary Table S1). The result was the same even in the presence of exogenous pheromone (see Supplementary Table S1). However, the *nlp-24* expression was slightly reduced in the *daf-22* mutants at 25 °C (Supplementary Fig. S4b). We immediately suspected that an abolished negative effect of pheromones on the *nlp-24* expression in *daf-22* mutants under starvation stress might be due to their pheromone sensing deficiency in addition to pheromone deficiency. The *daf-22* mutants exhibit deficiency in pheromone biosynthesis and abnormally high insulin activity (DAF-28), which might consequently result in a dauer formation defect^24^. Also, *daf-22(ok693)* mutants showed deficiency in pheromone sensing, which might be caused by massive accumulation of fats inside the body^25^. To test this suspicion, we measured the *nlp-24* expression in an ASK neuron-specific *daf-22* overexpression (*O/E*) line^24^. The *nlp-24* expression was upregulated in this *O/E* line at both 20 and 25 °C compared to the *daf-22* mutants (Supplementary Fig. S4c,d) (see Supplementary Table S1). These findings highlight the importance of the maintenance of intact pheromone metabolism (e.g., biosynthesis and sensing) for the negative regulatory effect of pheromones on opioid signaling.

To determine whether pheromones also negatively regulated opioid receptor-mediated signaling like they controlled the opioid ligand levels, we measured the *nlp-24* expression in *npr-17* mutants in the presence of a pheromone at 20 °C. As anticipated, the *nlp-24* expression was essentially unchanged at 20 °C with pheromone treatment (ascr#2) and was higher at 25 °C (Fig. 2b), independent of the nutritional state (Fig. 2c). These results confirm the previous finding that NLP-24 might autonomously act on NPR-17 as a ligand^4^, which possibly bases the coupling of food-seeking behavior and pharyngeal pumping in early starvation stage.

### Opioid signaling also negatively modulated the pheromone metabolism under starvation

**Figure 3:**
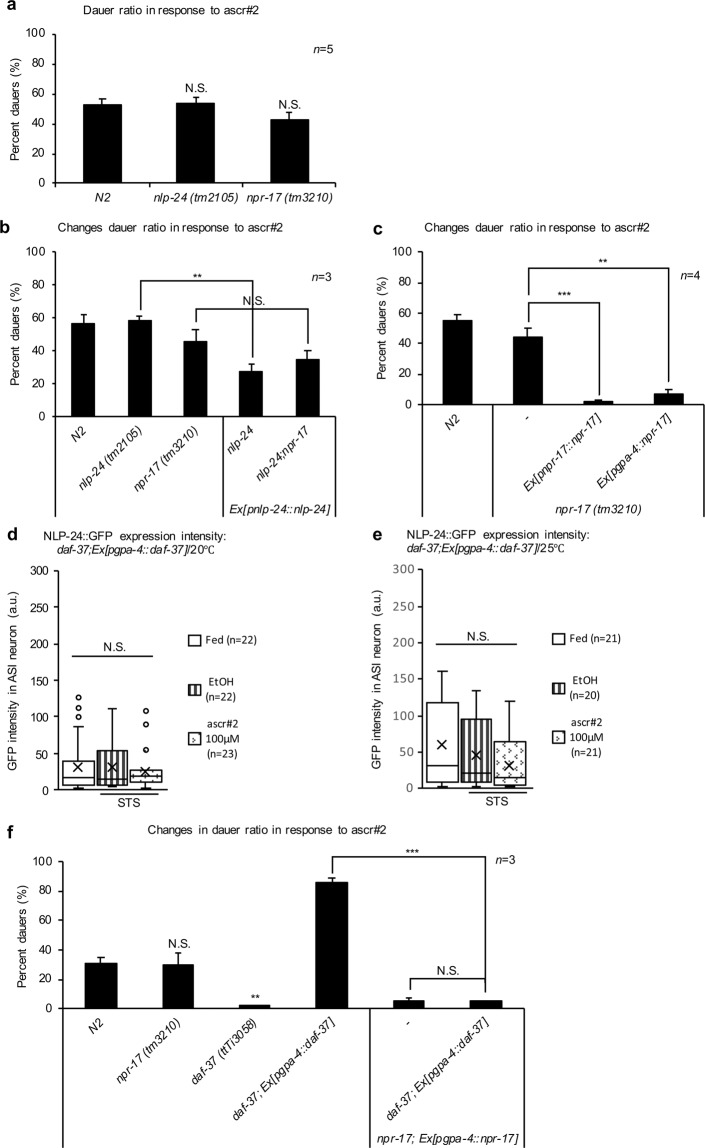
The relationship between opioid signaling and the ascaroside pheromone receptor in the ASI neurons. (**a–c**,**f**) The dauer formation ratios of **(a)** the *nlp-24(tm2105)* and *npr-17(tm3210)* worms (*n* = 5), **(b)** the *nlp-24*-overexpression lines, (*n* = 3), **(c)** the *npr-17*-overexpression lines (*n* = 4), and **(f)** the double transgenic line expressing *npr-17* and *daf-37* specifically in the ASI neurons. The dauer formation assay was performed by incubating eggs from each strain on plates containing 38 μM ascr#2 (>95.0% pure by HPLC) at 25°C for 3 days and then counting the dauer larvae. The data are represented as the mean with SEM from more than three different experiments with three technical repeats. *, **, and *** indicate *p-*values <0.05, <0.01, and <0.001, respectively. N.S. indicates no statistically significant difference. (**d,e**) The expression of NLP-24::GFP in the ASI neurons under fed, STS, and with ascr#2 conditions in the ASI-specific *daf-37* overexpression line (**d**) at 20 °C, and (**e**) at 25°C. Method and data processing for measured values are the same as in the legend of Fig. 1. Each box in the box plot was represents the inter-quartile range, the bar in the box represents median, and the X symbols represents the mean of the measured values under each condition. N.S. indicates no statistically significant difference. All *p-*values were calculated by unpaired *t-*test.

Because NPR-17 rather than NLP-24 negatively regulated pheromone biosynthesis (see Fig. 2a), we hypothesized that dauer formation might also be influenced by opioid signaling. To test this hypothesis, we analyzed the dauer formation ratio in *nlp-24(tm2105)* and *npr-17(tm3210)* mutants in the presence of a pheromone at 25 °C under optimal conditions^15,23,24^ (see Supplementary Fig. S2). The relative dauer formation ratio in the opioid mutants and N2 (wildtype) worms were similar (Fig. 3a). However, the dauer ratios (%) in the *nlp-24 O/E* and *npr-17 O/E* lines were significantly lower than in the wildtype worms (Fig. 3b,c), suggesting that the overexpression of opioid signaling genes antagonizes dauer formation during STS. Furthermore, the dauer formation ratio of the *npr-17* mutant carrying the *nlp-24 O/E* construct phenocopied that of the *npr-17* mutant (Fig. 3b). The dauer formation ratio in the ASI neuron-specific *npr-17 O/E* strain was also lower, similar to that of the *npr-17 O/E* strain (Fig. 3c).

To investigate whether ASI neuron-specific *nlp-24* expression was influenced by pheromone signaling, we measured the expression of an NLP-24::GFP level in an ASI neuron-specific *daf-37 O/E* strain^19^. The NLP-24 protein level was lower in this strain (Fig. 3d,e) compared to the control line (see Supplementary Table S1), which indicates that ASI neuron-specific *daf-37* overexpression might result in ascr#2 hypersensitivity^19^. We next performed a dauer formation assay with an ASI neuron-specific *O/E* strain to search for relationships among opioid and pheromone receptors. The ASI neuron-specific *daf-37* and *npr-17 O/E* resulted in the same phenotype observed upon ASI neuron-specific *npr-17 O/E* (Fig. 3f), under which conditions the highly elevated *nlp-24* expression detected during starvation was starkly reduced by pheromone signaling.

To explore possible connections among DAF-37-mediated pheromone sensing and opioid signaling in the ASK neurons, we examined *nlp-24* expression in an ASK neuron-specific *daf-37 O/E* line^19^. The *nlp-24* expression was lower in two independently-produced *O/E* lines compared to the *control line* (Supplementary Fig. S5) (see Supplementary Table S1). However, this effect could be due to decreased ASK neuron functionality rather than a direct effect exerted by pheromone sensing^24^. Based on these results, we conclude that opioid signaling had a negative effect on pheromone metabolism.

### GPA-3 played a key role in prioritizing opioid/pheromone signaling under starvation

**Figure 4:**
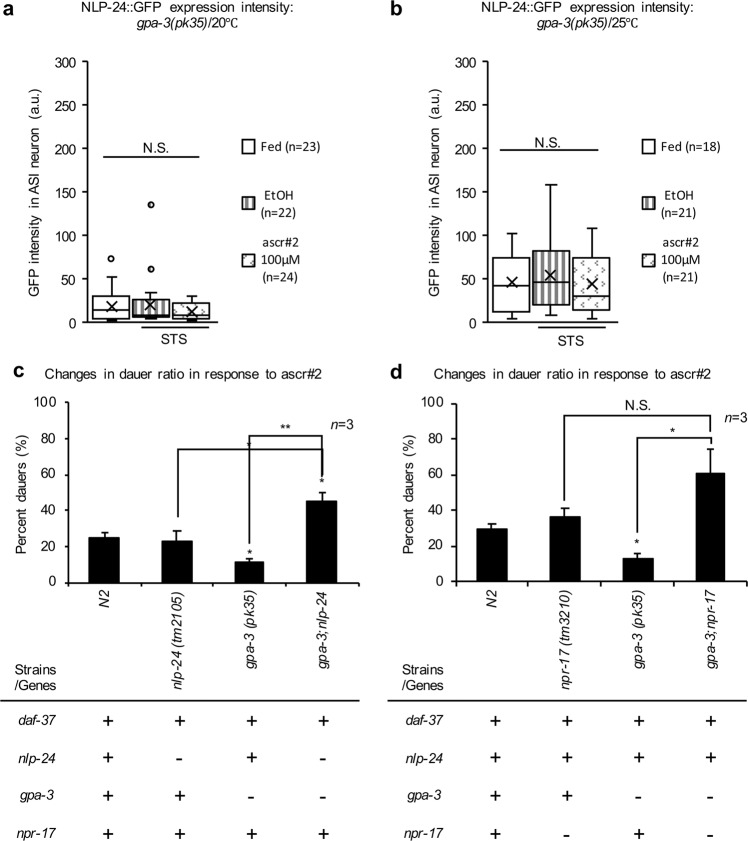
The relationship between opioid signaling and early ascaroside pheromone reception. (**a,b**) The expression of NLP-24::GFP in the ASI neurons under fed, starved, and starved with ascr#2 conditions in the *gpa-3(pk35)* background **(a)** at 20°C, and **(b)** at 25°C. Method and data processing for measured values are the same as in the legend of Fig. 1. Each box in the box plot was represents the inter-quartile range, the bar in the box represents median, and the X symbols represents the mean of the measured values under each condition. N.S. indicates no statistically significant difference. All *p-*values were calculated by unpaired *t-*test. **(c,d)** The dauer formation ratios of (**c)** the *nlp-24(tm2105);gpa-3(pk35)* double mutant worms (*n* = 3), and **(d)** the *npr-17(tm3210);gpa-3(pk35)* double mutant worms (*n* = 3). The method of dauer formation assay are the same as in the legend of Fig. 3. The data are represented as the mean with SEM of three different experiments with three technical repeats. *, **, and *** indicate *p-*values <0.05, <0.01, and <0.001, respectively. N.S. indicates no statistically significant difference. All *p-*values were calculated by unpaired *t-*test. The tables below present the included or excluded genes in each mutant strain.

As GPA-3 perceives the pheromone sensing signal during dauer formation^9,10,18,20,26^, we hypothesized that it could regulate the interaction of opioid signaling and pheromone signaling. To test this hypothesis, we measured the NLP-24 protein levels in pheromone-treated *gpa-3(pk35)* mutants at 20 °C under three treatment conditions: fed, starved (STS), and starved with pheromone. Notably, the NLP-24 protein level was very low in the *gpa-3* mutants that were maintained at 20 °C under all three conditions (Fig. 4a). An increase in temperature to 25 °C slightly stimulated the NLP-24 expression under the same growth conditions (Fig. 4b), but the addition of the pheromone had no effect on the NLP-24 expression in the *gpa-3* mutants (Fig. 4a,b). This result seems to mirror that for the *npr-17* mutant in which the *nlp-24* expression was essentially unchanged at 20 °C with pheromone treatment (ascr#2) under STS (see also Fig. 2b). This indicates that GPA-3 not only directly controls opioid signaling but also has a functional relationship with NPR-17, a ${{\rm{G}}}_{{\rm{o}}}$-coupled receptor^27,28^. Therefore, we wanted to investigate how GPA-3 involved in opioid signaling pathways affected dauer formation. Although the dauer ratios in the *nlp-24* mutants were quite similar to the N2 wildtype (left in Fig. 4c; see also Fig. 3a), but the *gpa-3;nlp-24* double mutants had a much higher dauer ratio compared to the dauer-defective *gpa-3* mutants (right in Fig. 4c). This result seems contradictory to our expectation that the double mutants in the dauer ratio might have phenocopied to the *gpa-3* mutant, but it also strengthens the idea that GPA-3 directly controls both pheromone (perception) and opioid signaling. Next, we wanted to know how the molecular interaction between *gpa-3* and *npr-17* affected dauer formation. The dauer ratio of the *npr-17* mutant was similar to the N2 wildtype, while the *gpa-3* mutant had a lower dauer ratio, as previously reported^9,26^. However, the *gpa-3;npr-17* double mutants had essentially the same phenotype as the *npr-17* single mutants (Fig. 4d). These results support the notion that GPA-3 might have dual regulatory functions for both opioid and pheromone signaling and thus could be able to interact with NPR-17 depending on stress duration (i.e., STS or LTS). It would be interesting to elucidate its potential mode of action for both signaling pathways under the different stress duration.

### Positive effects of insulin and serotonin on the opioid signaling under starvation

**Figure 5:**
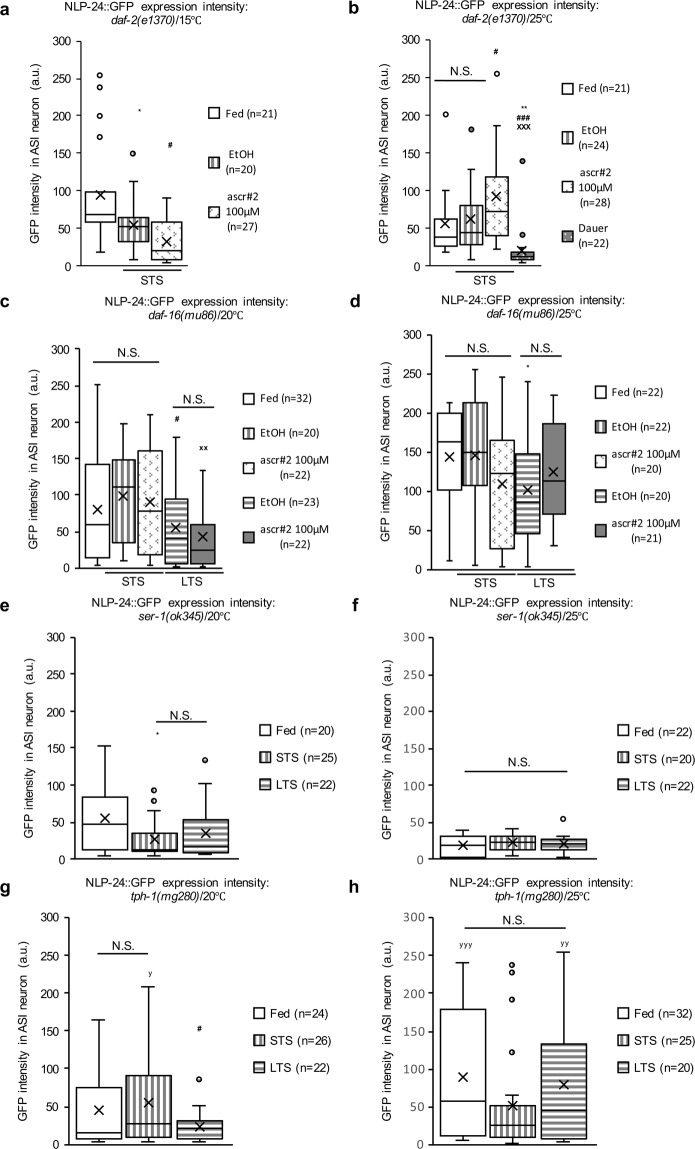
The effects of the insulin and serotonin signaling pathways on opioid-like signaling. (**a–h**) The expression of NLP-24::GFP in the ASI neurons under fed, starved, and starved with ascr#2 conditions **(a)** in the *daf-2(e1370)* background at 15°C, (**b)** at 25°C, **c** in the *daf-16(mu86)* background at 20°C, **(d)** at 25 °C, **(e)** under fed, and starved conditions in the *ser-1(ok345)* background at 20°C, **(f)** at 25°C, **(g)** in the *tph-1(mg280)* background at 20°C, **(h)** at 25°C. Method and data processing for measured values are the same as in the legend of Fig. 1. Each box in the box plot was represents the inter-quartile range, the bar in the box represents median, and the X symbols represents the mean of the measured values under each condition. *, **, and *** indicate *p-*values <0.05, <0.01, and <0.001, respectively, compared to the fed condition. #, ##, and ### indicate *p-*values <0.05, <0.01, and <0.001, respectively, compared to STS. x, xx, and xxx indicate *p-*values <0.05, <0.01, and <0.001, respectively, compared to STS with ascr#2 treatment (100μM). y, yy, and yyy indicate a *p-*value <0.05, <0.01, and <0.001, respectively, compared to the *ser-1(ok345)* background. N.S. indicates no statistically significant difference. All *p-*values were calculated by unpaired *t-*test.

Given the cross-negative influence between opioid and pheromone signals and because the insulin signaling pathway strongly affects pheromone-induced dauer formation^20,24,29–31^, we hypothesized that there might also be an interaction between insulin signaling and opioid signaling. To test this hypothesis, we first measured the *nlp-24* expression in the ASI neurons in *daf-2(e1370)* mutants, which lack a functional insulin receptor^7,29,32^. In the *daf-2* mutants, unlike in the control line, the *nlp-24* expression varied at 20 °C and 25 °C (Fig. 5a,b) (see Supplementary Table S1). However, in the *daf-2* mutant dauer worms, the *nlp-24* level was significantly lower (Fig. 5b). We detected almost no *nlp-24* expression in the intestine, regardless of the growth temperature or nutritional state (Supplementary Fig. S6). We checked whether *daf-16* was involved in *nlp-24* regulation^18,31^ using the *daf-16(mu86)* mutant, which was a null mutant of the *daf-16* gene and affected dauer formation^24,33,34^. The *nlp-24* expression level in the *daf-16(mu86)* mutants was essentially the same as the control strain, although there was no increase at 20 °C (Fig. 5c,d) (see Supplementary Table S1). These results indicate that insulin signaling positively modulated the opioid signaling pathway only during STS at 20 °C.

Next, we examined how serotonin signaling modulated the *nlp-24* expression under starvation stress. The expression of *ser-1* in the ASI neurons which encodes the serotonin receptor, is upregulated upon recognition of starvation cues^27^. We found low *nlp-24* expression levels in the *ser-1(ok345)* mutants that was independent of the starvation duration (Fig. 5e,f), suggesting that serotonergic signaling stimulated the *nlp-24* expression in ASI neurons. To confirm this possibility, we measured the *nlp-24* expression levels in *tph-1(mg280)* mutant worms, which do not synthesize serotonin^35^. At 20 °C, the *nlp-24* expression levels in the *tph-1(mg280)* and *ser-1(ok345)* mutant strains were similar (Fig. 5e,g). In contrast, the *nlp-24* expression level in the *tph-1(mg280)* mutant was higher than that in the *ser-1(ok345)* mutant at 25 °C, although it was consistent during starvation duration (Fig. 5f,h). Except for the starvation variable, the increase in *nlp-24* expression by these temperatures can be considered to be similar to that shown in the *control line*. (see Fig. 1e,f and Supplementary Table S1**)**. Based on these results, we concluded that *ser-1* (serotonin receptor), not *tph-1* (serotonin biosynthesis enzyme) that is not temperature-sensitive mutant, stimulates cell type-specific expression of *nlp-24* in the ASI neurons depending on the duration of the starvation stress and the growth temperature.

### Negative effects of SKN-1 and NHR-69 on the opioid signaling under starvation

**Figure 6:**
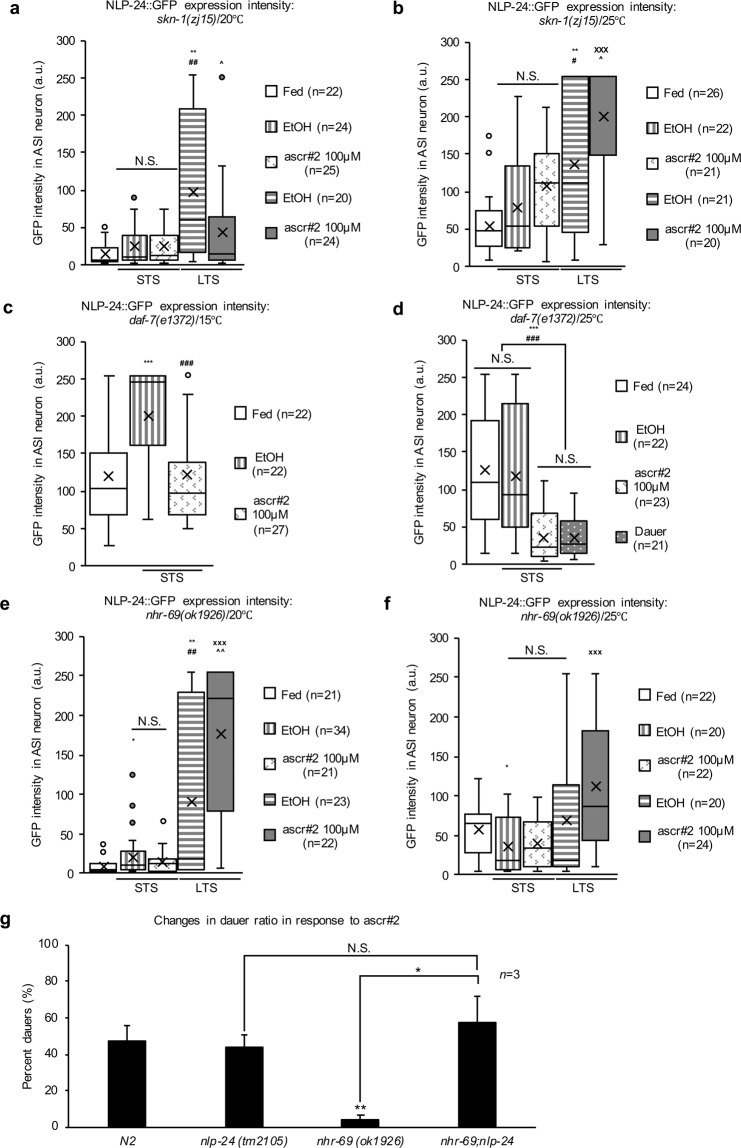
The effects of the *skn-1* and TGF-β pathways on opioid-like signaling. (**a–f**) The expression of NLP-24::GFP in the ASI neurons under fed, starved, and starved with ascr#2 conditions in the *skn-1(zj15)* background **(a)** at 20°C, **(b)** at 25°C, **(c)** in the *daf-7(e1372)* background at 15°C, **(d)** at 25°C, **(e)** in the *nhr-69(ok1926)* background at 20°C, and **(f)** at 25°C. Method and data processing for measured values are the same as in the legend of Fig. 1. Each box in the box plot was represents the inter-quartile range, the bar in the box represents median, and the X symbols represents the mean of the measured values under each condition. *, **, and *** indicate *p-*values <0.05, <0.01, and <0.001, respectively, compared to the fed condition. #, ##, and ### indicate *p-*values <0.05, <0.01, and <0.001, respectively, compared to the STS. x, xx, and xxx indicate *p-*values <0.05, <0.01, and <0.001, respectively, compared to STS with ascr#2 treatment (100μM). ^, ^^, and ^^^ indicate *p-*values <0.05, <0.01, and <0.001, respectively, compared to the LTS condition. N.S. indicates no statistically significant difference. All *p-*values were calculated by unpaired *t-*test. **(g)** The dauer formation ratio of the *nlp-24(tm2105);nhr-69(ok1926)* double mutant worms (*n* = 3). The method of dauer formation assay are same as in the legend of Fig. 3. The data are represented as the mean with SEM of three different experiments with three technical repeats. *, **, and *** indicate *p-*values <0.05, <0.01, and <0.001, respectively. N.S. indicates no statistically significant difference. All *p-*values were calculated by unpaired *t-*test.

The observed cross-negative influence between opioid and pheromone signaling suggests that transcription factors specifically expressed in the ASI neurons might play a role in molecular interaction between these two signals. To explore this possibility, we mined the modENCODE data (www.modencode.org) and found that the stress response transcription factor SKN-1 bound to the *nlp-24* promoter region during the L2 stage (Supplementary Fig. S7)^36,37^. Furthermore, the dauerDB data indicated that *skn-1* was highly expressed in the dauer stage (Supplementary Fig. S8)^21^. Previous work demonstrated that *skn-1* likely has four isoforms, two of which (*a* and *b*) are expressed *in vivo* in the ASI neurons and might be activated by starvation stress^38–40^. We used the *skn-1a/c* knockdown mutant allele *(zj15)*^41^ because the homozygous *skn-1* knockout is lethal^42^. We measured the NLP-24 protein level in the *skn-1* mutants and noticed that their *nlp-24* expression was very low during STS following pheromone treatment at 20 °C (*left* in Fig. 6a). However, the *nlp-24* expression was highly increased in the *skn-1* mutants under LTS at 20 °C (*right* in Fig. 6a), indicating that SKN-1 suppressed the *nlp-24* expression only during extended stress. Consistently, the addition of pheromones abolished the increased *nlp-24* expression in the *skn-1* mutants at 20 °C under LTS (*right* in Fig. 6a). Interestingly, at 25 °C the addition of pheromones stimulated the *nlp-24* expression in the *skn-1* mutants, and this effect was independent of the stress duration (Fig. 6b). Regardless of the incubation temperature (20 or 25 °C), the *nlp-24* expression was consistently low in the fed *skn-1* mutants (Fig. 6a,b). One interpretation of these results is that when the worms sensed the dauer-inducing temperature (25 °C), starvation and pheromone might have cooperated to suppress the *nlp-24* expression via the SKN-1 activation. This hypothesis is supported by previous report showing that worms carrying a gain-of-function *skn-1* mutation tended to enter the dauer stage more frequently than wildtype worms^43^. Thus, the suppressive effects of SKN-1 on the *nlp-24* expression likely depended on the temperature, duration of the starvation stress, and presence of pheromone.

In addition to insulin signaling, the pheromone-induced dauer formation is also regulated by TGF-β signaling^8,20^, as DAF-7 ligand expression is highly suppressed in the presence of pheromone^43,44^. Pharyngeal pumping is also influenced by food restriction and pheromone^45^. To understand the relationship between TGF-β signaling and the pheromone-dependent attenuation of opioid signaling, we measured the *daf-7* expression following pheromone treatment in *nlp-24*, *npr-17* mutants and wildtype animals. The pheromone treatment caused similar reductions in the *daf-7* levels in all three strains (Supplementary Fig. S9), suggesting that *daf-7* was not regulated by opioid signaling. To determine if there was an opposing epistatic interaction between TGF-β and opioid signaling, we measured the NLP-24 expression in the *daf-7*(*e1372*) mutants^46^ at different temperatures. While the NLP-24 level was increased at 15 °C under STS, there was no change at 25 °C under STS (Fig. 6c,d). The pheromone treatment caused similar reductions in the NLP-24 levels in both groups (Fig. 6c,d). Because the *daf-7* expression is suppressed by pheromone^44^, we wanted to examine if TGF-β-related transcription factors expressed in the ASI neurons might modulate the opioid signaling and dauer formation^47,48^. We tested this possibility by surveying the expression levels of the relevant transcription factors in the dauer stage using dauerDB^21^. The *nhr-69* expression was then elevated in the dauer stage, while the expression levels of the other transcription factors (DAF-3 and DAF-5) were suppressed (Supplementary Fig. S8). The NLP-24::GFP level was higher in the *nhr-69* mutants at 20 °C depending on the length of the exposure to starvation stress (Fig. 6e), which resembled the NLP-24 expression pattern in the *skn-1* mutants at 25 °C (Fig. 6b). The *nlp-24* expression in the *nhr-69* mutants at 25 °C (Fig. 6f) was similar to the *daf-*2 mutants at 25 °C (see Fig. 5b), suggesting that NHR-69 suppressed the *nlp-24* expression even when insulin signaling was disrupted^48^. We next wondered whether the *nhr-69*-dependent suppression of *nlp-24* could induce dauer formation. We performed dauer formation assays with *nhr-69;nlp-24* double mutants and *nlp-24* single mutants in the presence of ascr#2. The double mutants showed the same phenotype as the *nlp-24* single mutants, while the *nhr-69* mutants had a much lower dauer formation rate (Fig. 6g). Taken together, these results suggest that the TGF-β signaling likely enhanced the pheromone-mediated suppression of *nlp-24* expression, which could favorably drive dauer formation.

## Discussion

**Figure 7:**
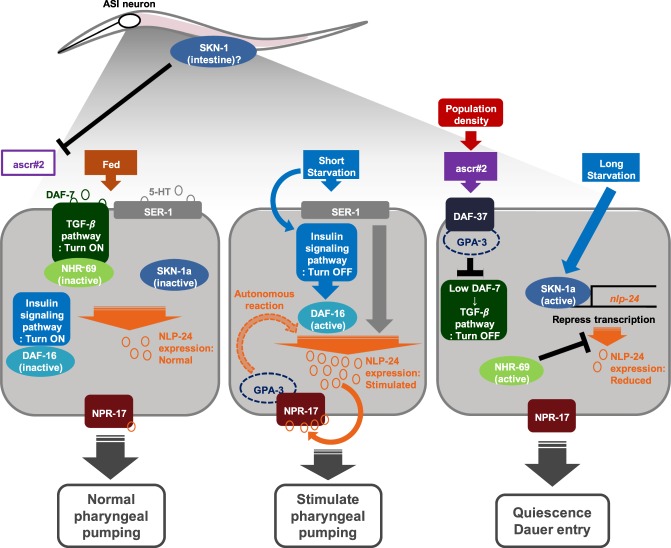
A proposed landscape of cross-antagonism between opioid and pheromone signaling under different growth conditions. Under a fed state (*left*), TGF-β, insulin, and serotonergic signaling might be in ‘active state’, which maintained the moderate expression levels of *nlp-24* and NPR-17, making normal pharyngeal pumping rate. Since DAF-16 and SKN-1 might not be activated, pheromone actions (e.g., ascr#2) were unlikely active. Under STS (*middle*), the opioid signaling mediated by activated *nlp-24* expression might occur autonomously with concomitant activation of pharyngeal pumping, which might also cause GPA-3 to interact with NPR-17. When encountered with sudden stress (e.g., increased population density) (*right*), pheromone ascr#2 would bind to seemingly DAF-37 (and GPA-3), which might subsequently suppress TGF-β signaling, but NHR-69, a downstream factor of TGF-β signaling, might be activated and then suppress the *nlp-24* expression (see also Fig. 6e,f). Under LTS, SKN-1a would be activated and then likely bound to one of putative promoter regions (e.g., binding region I, see also Supplementary Fig. S7) as a negative transcription factor. Together, this might culminate in attenuation of the opioid signaling but stimulation of the pheromone-mediated dauer entry to facilitate stress avoidance.

To the best of our knowledge, this is the first report of a molecular interaction between two anti-stress signals—opioids and pheromones—that facilitate stress avoidance in response to different durations of stress exposure in early stage *C. elegans* larvae (L2d stage). The nature of the interaction between the opioid and pheromone signaling appears to be “cross-antagonistic,” or “competitive.” The interaction involves several key regulators of chemosensory processes (e.g., GPA-3, DAF-37, NPR-17), metabolic pathways (e.g., insulin, serotonin, TGF-β), and anti-oxidation (e.g., SKN-1) that are actively interlinked (Fig. 7). This interaction process also requires a specific stress duration (STS vs. LTS) and additional physiological setting (e.g., temperatures, ASI neurons). Note that as food becomes scarce during LTS, the competition between two seemingly opposing signaling pathways, opioid and pheromone, weakens. More importantly, GPA-3 and SKN-1 are proposed to function as key regulators in this process, which likely involves a diverse protein-protein interaction depending on the duration of starvation.

One of the notable findings of this study relates to potential dual regulatory roles of GPA-3 not only controlling the well-known pheromone perception leading to dauer formation^9,18,26^ but also modulating opioid signaling under starvation (see Fig. 4). For instance, GPA-3 appeared to act as a switchboard to prioritize opioid/pheromone signaling depending on the duration of starvation. This proposed switchboard function of GPA-3 may be attributed to its predicted selective binding property to either NPR-17 under STS, or DAF-37 under LTS (Supplementary Table S2)^19,27,49^. That is, as the *nlp-24* concentration increases under STS, GPA-3 could interact with NPR-17, preventing GPA-3 contact with DAF-37 for dauer formation, while maintaining roaming behavior (middle in Fig. 7). Under LTS, however, the opposite direction seemed to be the case. Data mined from dauerDB showed that in dauer larvae, the expression of both *gpa-3* and *daf-37* was increased, but *npr-17* was suppressed (Supplementary Fig. S10). This might possibly cause GPA-3 to preferentially interact with DAF-37 to enhance pheromone binding for dauer formation under LTS. The exact mode of such binding remains to be elucidated in the future.

Among the transcription or modulatory factors associated with the opioid-pheromone interaction under LTS, SKN-1a, a stress-response transcription factor, seems to play a pivotal role in stress avoidance. Two possible mechanisms can be considered. The first involves the activation of SKN-1a. Note that under a fed state, SKN-1 was usually inactive and did not stimulate pheromone biosynthesis (Supplementary Fig. S11), confirming a stress-specific function of SKN-1. However, upon the worms’ recognition of the LTS cues, SKN-1a was activated and thus suppressed the *nlp-24* expression (see Fig. 6b). Consequently, this activated SKN-1a likely bound to the predicted promoter region of the *nlp-24* gene (e.g., binding region **I**, −1697 to −1289) (see Supplementary Fig. S7) as a negative transcription factor during the L2 stage (right in Fig. 7) as previously reported^36,37^. This might suppress the *nlp-24* gene expression and attenuated both the opioid signaling and pharyngeal pumping (right in Fig. 7). The second possible mechanism involves the activation of *acox-1*: SKN-1 is known to activate *acox-1* in the starvation state^50,51^. This enzyme catalyzes the first reaction in the biosynthesis of the fatty acid component of pheromones^52^, and thus more pheromones would be produced by activated *acox-1* under LTS^50^ and could induce dauer formation. It would be interesting to investigate how SKN-1 binds to the promoter of *nlp-24* under LTS to facilitate stress avoidance.

Because the nematode *C. elegans* offers many advantages as a model organism for the study of neurobiology and neurodegenerative diseases^53^, our current work suggests the usefulness of opioid-overexpressing transgenic *C. elegans* strains for studying opioid overdose. These animals could potentially mimic the opioid overdose conditions that occur in vertebrate animals^13,54^. Overdoses from pain killers (e.g., opioid drugs) are a serious problem and have reached epidemic proportions as the number of opioid addicts continues to increase. Opioid overdoses cause more than 70,000 deaths per year in the United States (https://www.cdc.gov/nchs/nvss/vsrr/drug-overdose-data.htm). Given that our opioid-overexpressing transgenic animals showed a partial defect in dauer formation, which is thought to mimic an opioid hypersensitive state (see Fig. 3b,c), they could be useful as an animal model for the *in vivo* screening of potential antagonists for opioid expression. In fact, *C. elegans* has already been demonstrated to study chemical antagonists against cocaine and methamphetamine exposure, and these experiments yielded similar findings to those obtained using place conditioning with rats and mice^55,56^. Furthermore, *C. elegans* and vertebrates share a conserved opioid system^4^, the effects of pheromone on the expression of *nlp-24*, which encodes a β-endorphin-homolog, suggest possible approaches for modulating opioid drug function via pheromone mimetics and preference via manipulation of genetic or biochemical conditions. Since *C. elegans* NPR-17 is known to be activated by mammalian MOR-1 (μ-opioid receptor) and KOR (kappa-opioid receptor) agonists but such responses were abolished by naloxone^4^, it could thus be feasible to test the validity of such a model by measuring the effects of pretreatment with pheromone or pheromone mimetics on opioid preference in opioid-overexpressing animals (e.g., *nlp-24 O/E, npr-17 O/E*).

## Materials and Methods

### C. elegans strains and culturing

C*. elegans* strains (e.g., N2 Bristol, wild-type) were cultured using standard techniques^57^. We used the following worm strains in this study: N2 Bristol (wild-type), *daf-22(ok693), daf-22(ok693);* E*x[psrbc-64::daf-22, rol-6], daf-37(ttTi3058), daf-37(ttTi3058);* E*x[pgpa-4::daf-37], daf-37(ttTi3058);* E*x[psrbc-64::daf-37], gpa-3(pk35), nhr-69(ok1926), nlp-24(tm2105), npr-17(tm3210), gpa-3(pk35);nlp-24(tm2105), gpa-3;npr-17(tm3210), nhr-69(ok1926);nlp-24(tm2105), daf-7::*GF*P, nlp-24(tm2105);pdaf-7::*GF*P, npr-17(tm3210);pdaf-7::*GF*P, nlp-24(tm2105);* E*x[pnlp-24::nlp-24::SL2::*GF*P,myo-3::m*C*herry], daf-2(e1370);nlp-24(tm2105);* E*x[pnlp-24::nlp-24::SL2::*GF*P,myo-3::m*C*herry], daf-7(e1372);nlp-24(tm2105);* E*x[pnlp-24::nlp-24::SL2::*GF*P, myo-3::m*C*herry], daf-16(mu86);nlp-24(tm2105);* E*x[pnlp-24::nlp-24::SL2::*GF*P, myo-3::m*C*herry], daf-22(ok693);nlp-24(tm2105);* E*x[pnlp-24::nlp-24::SL2::*GF*P, myo-3::m*C*herry], daf-37(ttTi3058);nlp-24(tm2105);* E*x[pnlp-24::nlp-24::SL2::*GF*P, myo-3::m*C*herry], daf-37(ttTi3058);nlp-24(tm2105);* E*x[pnlp-24::nlp-24::SL2::*GF*P, myo-3::m*C*herry];* E*x[pgpa-4::daf-37], daf-37(ttTi3058);nlp-24(tm2105);* E*x[pnlp-24::nlp-24::SL2::*GF*P, myo-3::m*C*herry];* E*x[psrbc-64::daf-37], gpa-3(pk35);nlp-24(tm2105);* E*x[pnlp-24::nlp-24::SL2::*GF*P, myo-3:: m*C*herry], nhr-69;(ok1926);nlp-24(tm2105);* E*x[pnlp-24::nlp-24::SL2::*GF*P, myo-3:: m*C*herry], npr-17;(tm3210);nlp-24(tm2105);* E*x[pnlp-24::nlp-24::SL2::*GF*P, myo-3::m*C*herry], ser-1(ok345);nlp-24(tm2105);* E*x[pnlp-24::nlp-24::SL2::*GF*P, myo-3::m*C*herry], skn-1(zj15);nlp-24(tm2105);* E*x[pnlp-24::nlp-24::SL2::*GF*P, myo-3::m*C*herry], tph-1(mg280);nlp-24(tm2105);* E*x[pnlp-24::nlp-24::SL2::*GF*P, myo-3::m*C*herry], npr-17(tm3210); pdaf-7::*GF*P, npr-17(tm3210);* E*x[Pnpr-17::npr-17::*GF*P, Punc-122::R*F*P], npr-17(tm3210);* E*x[Pgpa-4::npr-17::SL2::R*F*P, Punc-122::*GF*P]*, and *daf-37(ttTi3058);npr-17(tm3210);* E*x[Pgpa-4::npr-17::SL2::R*F*P, Punc-122::*GF*P];* E*x[pgpa-4::daf-37]*.

The *daf-37(ttTi3058), daf-37(ttTi3058);* E*x[pgpa-4::daf-37]*, and *daf-37(ttTi3058);* E*x[psrbc-64::daf-37]* strains were received from Don-ha Park^19^, and the N2*;* E*x[pnlp-24::nlp-24::SL2::*GF*P, myo-3::m*C*herry], npr-17(tm3210);* E*x[Pnpr-17::npr-17::*GF*P, Punc-122::R*F*P]*, and *npr-17(tm3210);* E*x[Pgpa-4::npr-17::SL2::R*F*P, Punc-122::*GF*P]* strains were received from Mi Cheong Cheong^4^. The *daf-22(ok693);* E*x[psrbc-64::daf-22,rol-6]* strain was received from Saeram Park^24^. The other single mutant strains were received from the C*aenorhabditis* Genetics Center (CGC) and from the Mitani lab. The other double mutant strains and transgenic lines were produced in our laboratory and were confirmed with genotyping primers (Listed in Supplementary Table S3). Worms were grown on NGM seeded with E*. coli* OP50 as a food source.

### Dauer formation assay

Ascaroside pheromone 2 (ascr#2 or daumone 2) was chemically synthesized in our laboratory^15,22^. The ascr#2 was prepared by adding 1.0 ml of ethanol to the stock solution. The optimal dauer formation assay with varied concentrations was determined as described in previous studies^15,23,24,52,58^. For this assay, we used 38 μM ascr#2 (95.0% pure by HPLC)-supplemented fresh NGM (without peptone) plates seeded with 160 μg of heat-killed OP50. The dauer ratio was calculated by dividing the number of dauer larvae by the total number of worms. Each experiment was performed three or more times with triplicate plates.

### Measurement of NLP-24::GFP fluorescence

For this experiment, unless otherwise specified, the same NLP-24 translational reporters (e.g., Pnlp-24::nlp-24::GFP, NLP-24 fused with mCherry, NLP-24::SL2::GFP etc.) were used as previously described^4^. This experiment was also designed based on two previous studies^4,43^. For late L1 worm preparation, we prepared OP50-containing NGM plates which were laid ten adult NLP-24::GFP transgenic line worms and incubated them for 6 hours for obtaining eggs. After incubation, the adult worms were removed, and the eggs on the plates were incubated for 24 hours. We prepared these late L1 larvae^59^ and washed them with M9 buffer to remove the OP50. Next, we suspended the larvae in M9 buffer with ethanol as a vehicle to produce the starvation sample and in M9 buffer with ascr#2 for the ascr#2-supplemented sample. The ascr#2 concentration varied in different experiments. After the conditioning step, we added 50 mM sodium azide to the samples, incubated them for 5 min, and then transferred the worms to agarose pads on glass slides for imaging. The slides were imaged using an LSM880 confocal microscope (ZEISS, Jena, Germany) using a 40x water-immersion lens with FITC (to detect NLP-24::GFP) and rhodamine (to detect *myo-3::m*C*herry*) filters. All of the images were 8-bit and were analyzed using ZEN Black software (ZEISS). After imaging each sample, the maximum values of the GFP fluorescence for the regions of the images containing the ASI neurons were quantified using ImageJ. Because 8-bit images were analyzed, the measured values were recorded as numbers between 0 and 255. We compiled the measured values for each sample.

### Measurement of DAF-7::GFP fluorescence

We performed this experiment based on a previous study^44^. Late L1 larvae^59^ of the *daf-7*::GFP transgenic lines were prepared, washed, and then incubated overnight in M9 buffer alone or in M9 buffer containing 100 μM ascr#2. The sample imaging was performed the same way as the NLP-24::GFP imaging using a LSM880 confocal microscope (ZEISS, Jena, Germany).

### Measurement of pharyngeal pumping rates

We designed this experiment based on previous study^4^. C*ontrol line* worms (*nlp-24;*E*x[pnlp-24::nlp-24::*GF*P]*) were prepared and conditioned in same ways as measurement of NLP-24::GFP experiment. Each worm laid at NGM (without peptone) plates and dried. We counted pharyngeal grinder for 1 minute in each individual worm using SZX7 zoom stereo microscope at 56x magnification (Olympus, Tokyo, Japan). We used red fluorescence filter to confirm the *myo-3::m*C*herry* which was fluorescence marker in *control line*.

### Chemicals and reagents for ascaroside pheromone quantification

Analytical-grade ammonium acetate and formic acid were obtained from Sigma-Aldrich Chemical (St. Louis, MO, USA). Methanol, HPLC-grade water, and acetonitrile were supplied by Burdick & Jackson (Muskegon, MI, USA). Chemically synthesized ascr#1, 2, and 3, which were used as standards for the generation of calibration curves and as quality control (QC) samples, were prepared as previously described^52,58^.

### Extraction of pheromones from worm bodies and liquid culture samples

Frozen 20 μl samples containing 40 worms were thawed at ambient temperature, and 40 μl of methanol and 40 μl of IS working solution were added and mixed with a vortexer for several seconds. The samples were then mixed with 100 μl of 0.1% (v/v) formic acid in acetonitrile for 30 min using a vortex mixer (Thermomixer compact, Eppendorf, Hamburg, Germany). The acidic supernatant was dried in a Speed-Vac centrifuge (ScanVac ScanSpeed40, LABOGENE, Korea). Before analysis, the samples were reconstituted in 50 μl of 0.1% formic acid in 50% (v/v) acetonitrile and centrifuged at 13000xg for 10 min. For measuring, 2 μl was injected into the UPLC-tandem mass spectrometry (MS/MS) system.

### Liquid chromatography/Mass spectrometry

Separation of analytes by HPLC was performed on a C18 column (Imtakt Unison UK-C18 HPLC Column; 3 μm, 2.0 × 75 mm) at 30 °C using an Agilent 1200 HPLC system. The gradient conditions were as follows: mobile phase A, 0.1% (v/v) formic acid in 2 mM ammonium acetate buffer; mobile phase B, 0.1% formic acid in acetonitrile; 3.45% B for 11 min, followed by washing for 2 min in 97% B and re-equilibration in 3% B for 2 min. All analytes were eluted at a flow rate of 400 μl/min. Mass-spectrometric detection was performed on an API-4000 triple-quadrupole mass spectrometer (MDS SCIEX, Toronto, Canada) operated in MRM mode, using unit resolution on quadrupoles Q1 and Q3 as previously described^58^.

### Statistical analysis

Microsoft Excel was used to produce the graphs and to perform the statistical analyses. In the dauer formation assays, each dauer ratio in one trial represents the mean of the ratios on triplicate plates. We represented the mean ratios with the SEMs of each experiment in bar graphs from the individual trials. The *p-*values were calculated using unpaired *t-*test. For the box plots of NLP-24::GFP intensity, we calculated the mean for each condition by measuring individual worms. The exact mean and SEM values of various condition were summarized at Table. S1. The *p-*values were also calculated by unpaired *t-*test. For the quantification of ascr, each set was performed in triplicate with five identical individual samples. We calculated the mean value for each strain by performing three independent experiments. We normalized each measured value that of N2, which was set to 1. The means, SEMs and *p-*values were calculated from the normalized values. The *p-*values were calculated by unpaired *t-*test.

## Supplementary information

Supplementary Information
