# Supplementary material for: A novel functional cross-interaction between opioid and pheromone signaling may be involved in stress avoidance in Caenorhabditis elegans
Source: Sci Rep. 2020 May 5;10:7524. doi: 10.1038/s41598-020-64567-3 (PMC7200713; doi:10.1038/s41598-020-64567-3)
Supplement: Supplementary file 1 — Supplementary Information [file 41598_2020_64567_MOESM1_ESM.pdf]

Supplementary Information

A novel functional cross-interaction between opioid and pheromone signaling may be involved in stress avoidance in *Caenorhabditis elegans*

Jun Young Park[1,2], Mi Cheong Cheong[3], Jin-Young Cho[2], Hyeon-Sook Koo,[4] and Young-Ki Paik[1,2,*]

[1]Interdisciplinary Program in Integrative Omics for Biomedical Science, Yonsei University, Seoul 03722, Korea, [2]Yonsei Proteome Research Center, Yonsei University, Seoul 03722, Korea, [3]Department of Pharmacology, UT Southwestern Medical Center at Dallas, Dallas, TX 75390, USA, [4]Department of Biochemistry, College of Life Science and Biotechnology, Yonsei University, Seoul 03722, Korea

*Corresponding author: paikyk@yonsei.ac.kr or paikyk@gmail.com

Supplementary Figures S1-11
Supplementary Tables S1-3
Supplementary References

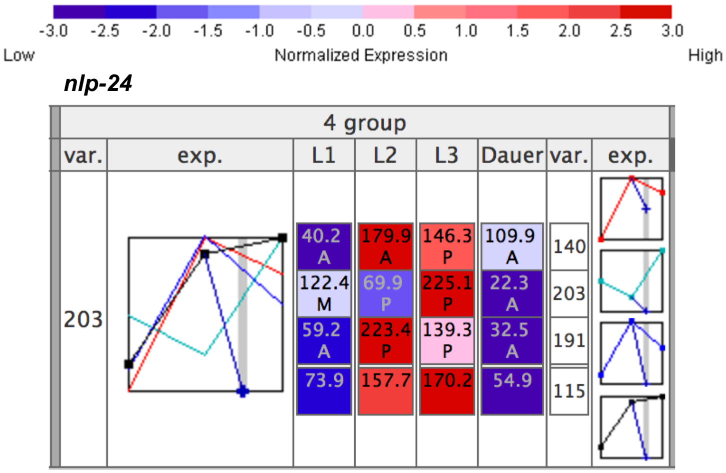

Supplementary Fig. S1. The *nlp-24* expression pattern obtained from dauerDB. Expression of *nlp-24*, the key ligand in the opioid-like signaling pathway, shows a significant reduction in the dauer state (taken from www.dauerdb.org). Details are described in the manual of this database at http://www.dauerdb.org/090202_Dauerdb_manual_v1.0.pdf. Gene expression of *nlp-24* was measured for 4-group worms at L1~L3 and dauer maintained on broth culture[1].

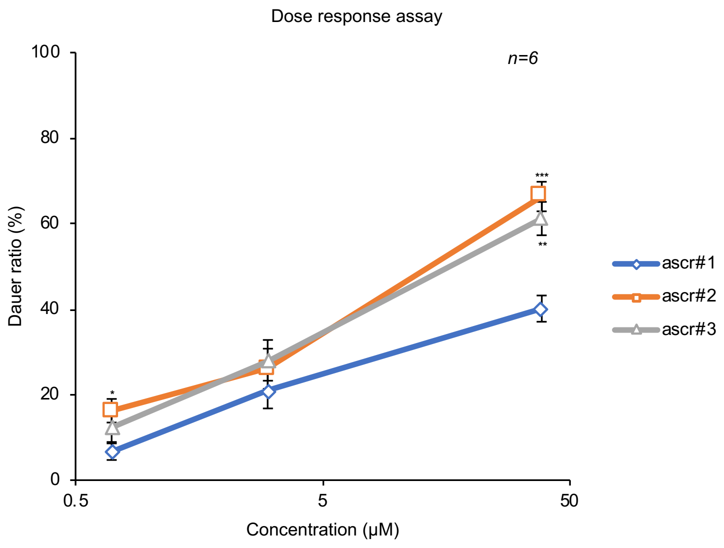

Supplementary Fig. S2. Dose response assay of wildtype worms using ascaroside pheromones. The dauer formation assay was performed by incubating eggs from each strain on plates containing 700 nM, 3 μM, and 38 μM of ascr#1, 2, and 3 at 25°C for 3 days and then counting the dauer larvae. The data are represented as the mean with SEM from more than six different experiments with three technical repeats. *, **, and *** indicate p-values < 0.05, < 0.01, and < 0.001, respectively, compared to the dauer ratio which treat ascr#1 in each concentration. All p-values were calculated by unpaired t-test.

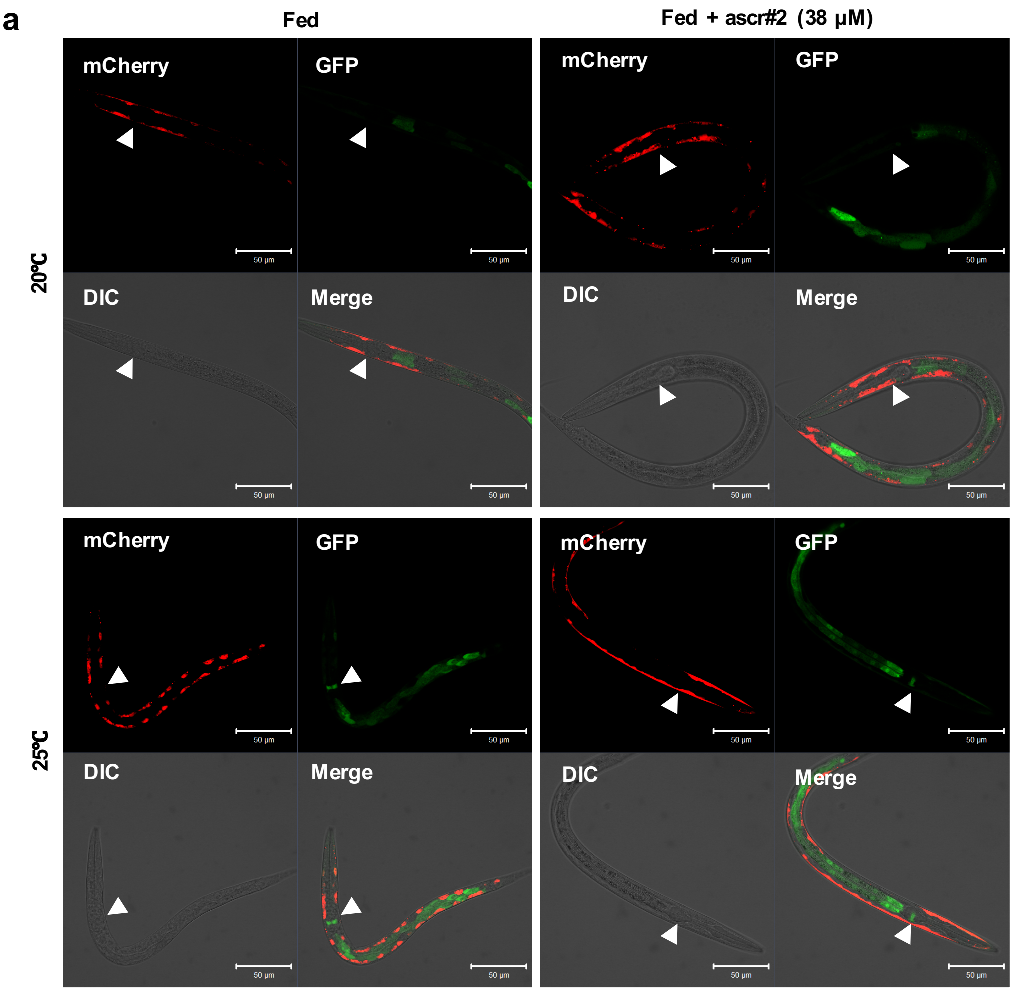
a
Fed
Fed + ascr#2 (38 μM)
mCherry
GFP
DIC
Merge
20℃
25℃

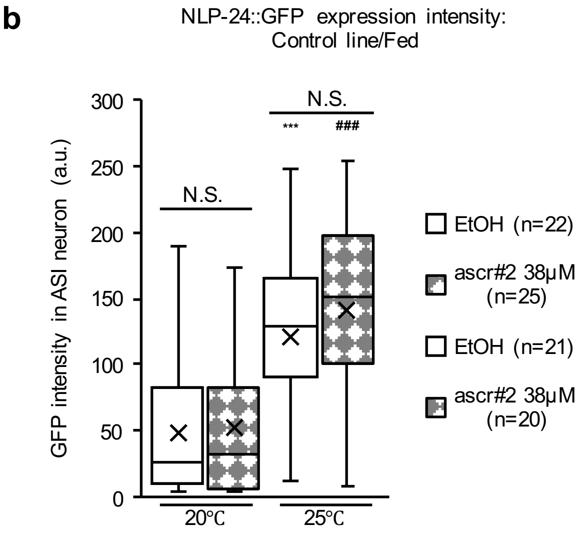
b
NLP-24::GFP expression intensity:
Control line/Fed
GFP intensity in ASI neuron (a.u.)
300
250
200
150
100
50
0
N.S.
N.S.
***
###
20℃
25℃
EtOH (n=22)
ascr#2 38μM (n=25)
EtOH (n=21)
ascr#2 38μM (n=20)

Supplementary Fig. S3. Changes in *nlp-24* expression in response to ascaroside pheromone under fed conditions. (a) The NLP-24::GFP expression was in the ASI neurons under fed conditions with the addition of ascr#2 (at 20°C and 25°C). The images were taken using a Zeiss LSM880 confocal microscope with a 40x water-immersion lens under each condition. A white arrow indicates the position of the ASI neurons. Bar 50 μm. **(b)** The NLP-24::GFP expression in the ASI neurons under the fed condition and in the presence of ascr#2 (at 20°C and 25°C). Each box plot was generated by using ImageJ to analyze the GFP fluorescence intensities in the ASI neurons in the 8-bit images taken under each condition (values from 0 ~ 255). Each measured value (in arbitrary units, a.u.) from the individual worms constituted a box plot specific for each condition. Each box in the box plot was represents the inter-quartile range, the bar in the box represents median, and the X symbols represents the mean of the measured values under each condition. *, **, and *** indicate *p*-values < 0.05, < 0.01, and < 0.001, respectively, compared to the fed condition. #, ##, and ### indicate *p*-values < 0.05, < 0.01, and < 0.001, respectively, compared to the fed condition with ascr#2 (38 μM). N.S. indicates no statistically significant difference. All *p*-values were calculated by unpaired *t*-test.

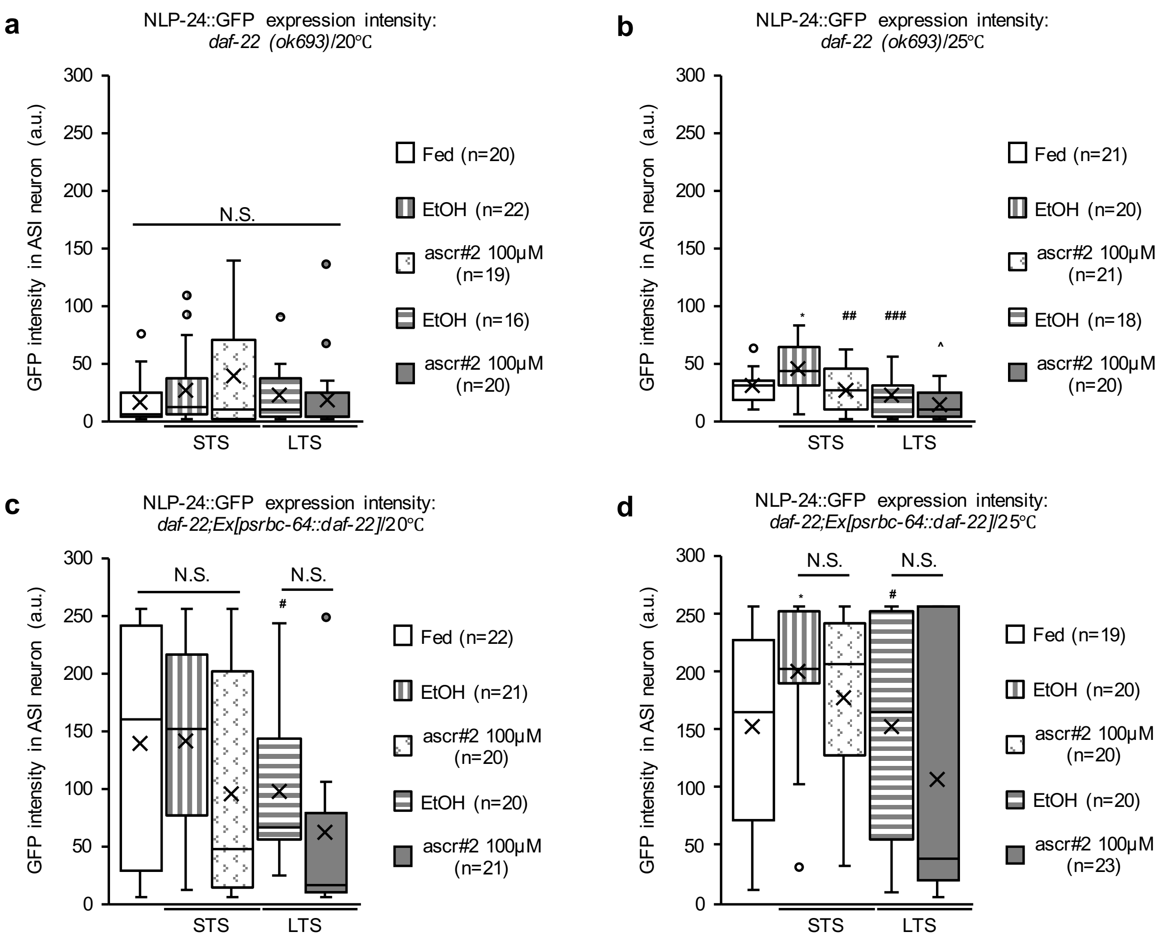

Supplementary Fig. S4. The relationship between *daf-2*2 and opioid signaling. (a-d) The expression of NLP-24::GFP in the ASI neurons under fed, starved, and starved with ascr#2 conditions in the *daf-22(ok693)* background (**a**) at 20°C, (**b**) at 25°C, (**c**) in the ASK-specific *daf-22* overexpression line at 20°C, and (**d**) at 25°C. Each box plot was generated by using ImageJ to analyze the GFP fluorescence intensities in the ASI neurons in the 8-bit images taken under each condition (values from 0 ~ 255). Each measured value (in arbitrary units, a.u.) from the individual worms constituted a box plot specific for each condition. Each box in the box plot was represents the inter-quartile range, the bar in the box represents median, and the X symbols represents the mean of the measured values under each condition. *, **, and *** indicate *p*-values < 0.05, < 0.01, and < 0.001, respectively compared to the fed condition. #, ##, and ### indicate *p*-values < 0.05, < 0.01, and < 0.001, respectively, compared to the STS.

^, ^^, and ^^^ indicate p-values < 0.05, < 0.01, and < 0.001, respectively, compared to the LTS. N.S. indicates no statistically significant difference. All p-values were calculated by unpaired t-test.

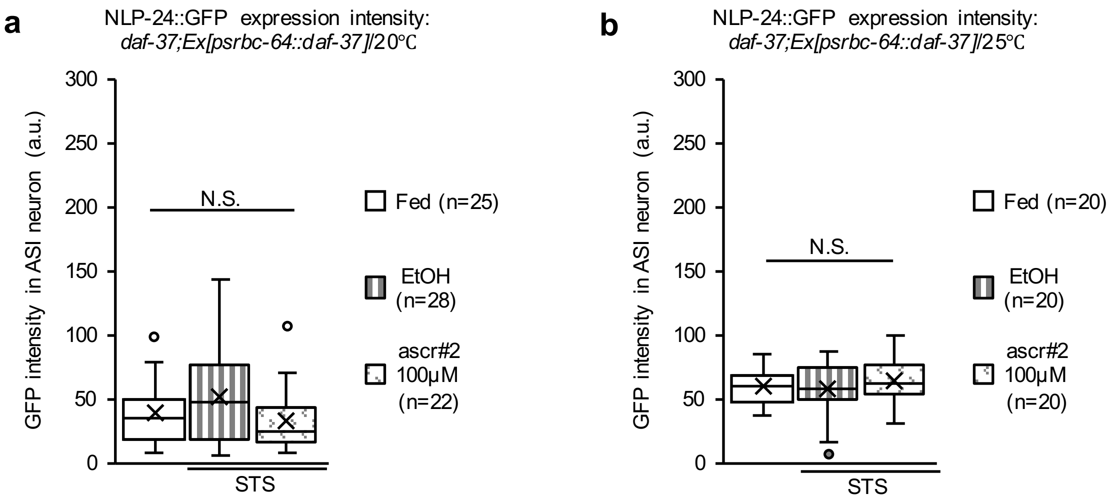

Supplementary Fig. S5. The relationship between opioid signaling and ascaroside pheromone signaling in the ASK neurons. (a, b) The expression of NLP-24::GFP in the ASI neurons under fed, starved, and starved with ascr#2 conditions in the ASK-specific *daf-37* overexpression line (**a**) at 20°C, and (**b**) at 25°C. Each box plot was generated by using ImageJ to analyze the GFP fluorescence intensities in the ASI neurons in the 8-bit images taken under each condition (values from 0 ~ 255). Each measured value (in arbitrary units, a.u.) from the individual worms constituted a box plot specific for each condition. Each box in the box plot was represents the inter-quartile range, the bar in the box represents median, and the X symbols represents the mean of the measured values under each condition. N.S. indicates no statistically significant difference. All *p*-values were calculated by unpaired *t*-test.

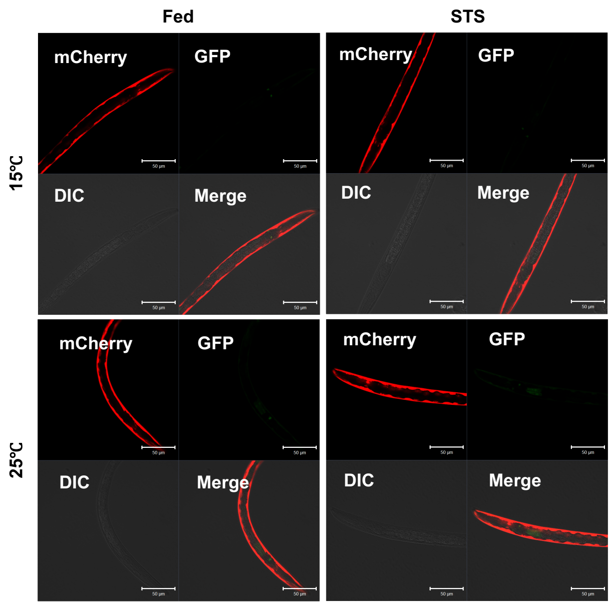

Supplementary Fig. S6. The expression of *nlp-24* in *daf-2(e1370)* mutant animals. The expression of NLP-24::GFP under fed and starved conditions in the *daf-2(e1370)* background. The images were taken using a Zeiss LSM880 confocal microscope with a 40x water-immersion lens under each condition. Bar 50 µm.

a

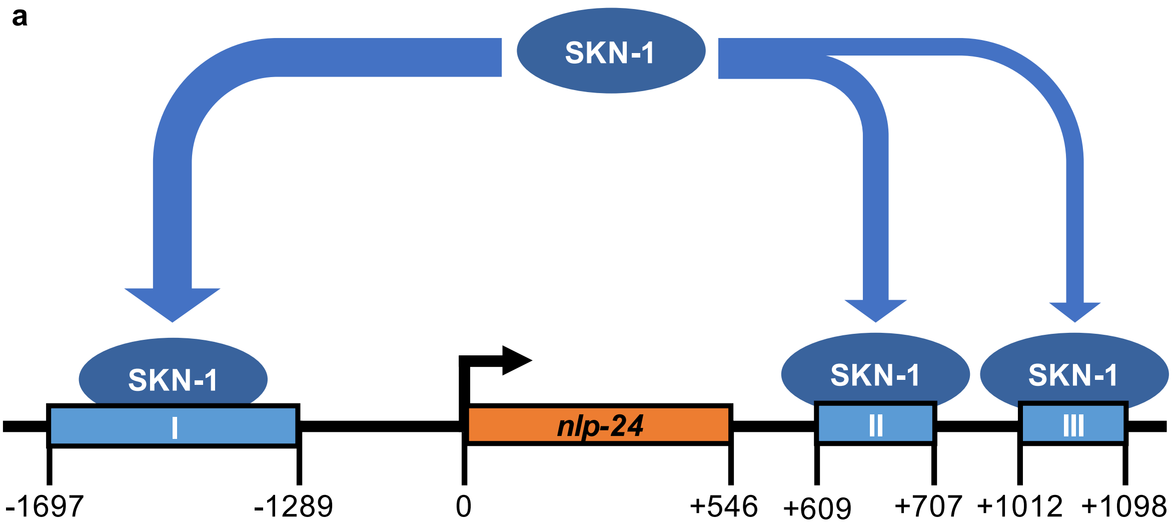

b

Binding Site	Position	Length	q-value
I	V:11614586..11614994	409	1.59327702107917e-07
II	V:11616892..11616990	99	0.000134442980015836
III	V:11617295..11617381	87	0.00251662782557436

Supplementary Fig. S7. The predicted binding mode of SKN-1 within the promoter region or intergenic region of *nlp-24* gene taken from the modENCODE RNAseq data. (a) Modified Supplementary Figure of the modENCODE RNAseq data about SKN-1 binding sites nearby the *nlp-24* gene region in the L2 stage. Shown is the potential SKN-1 binding sites located in the *nlp-24* gene. Orange box is coding region of *nlp-24* gene and blue boxes (I-III) are predicted binding regions of SKN-1. Each number under the sites indicate nucleotide distance from starting point of *nlp-24* gene. (**b)** Information of SKN-1 binding sites. There are three potential binding sites among which region I may likely be the main site according to q-values representing transcription factor binding affinity. This Supplementary Figure was redrawn from the data present in modENCODE database at www.modencode.org[2,3].

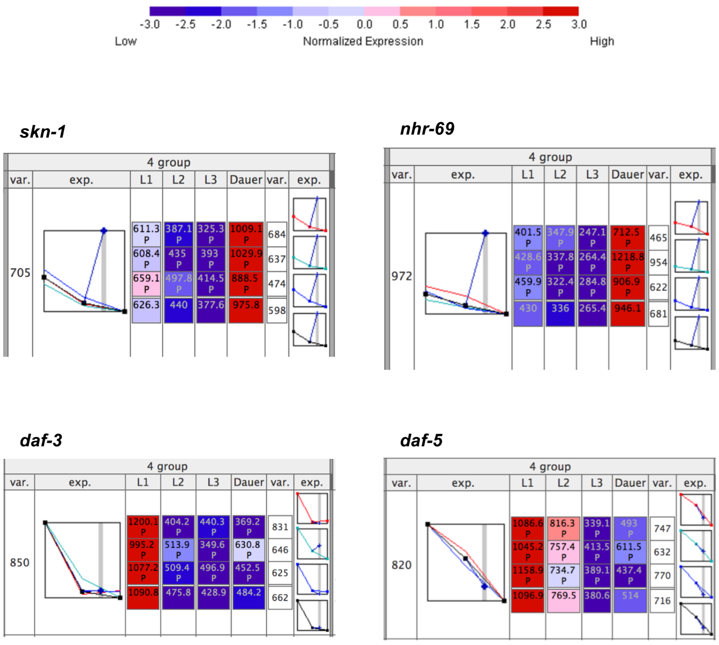

Supplementary Fig. S8. Growth stage-dependent expression patterns of *skn-1* and TGF-β pathway-related factors from dauerDB. This Supplementary Figure shows that the expression of *skn-1*, the gene encoding a starvation-related transcription factor, and *nhr-69*, the gene encoding a transcription factor in the TGF-β pathway, in dauer larvae. The expression of *daf-3* and *daf-5*, genes encoding other transcription factors in the TGF-β pathway is also seen in dauer larvae (taken from www.dauerdb.org) [1].

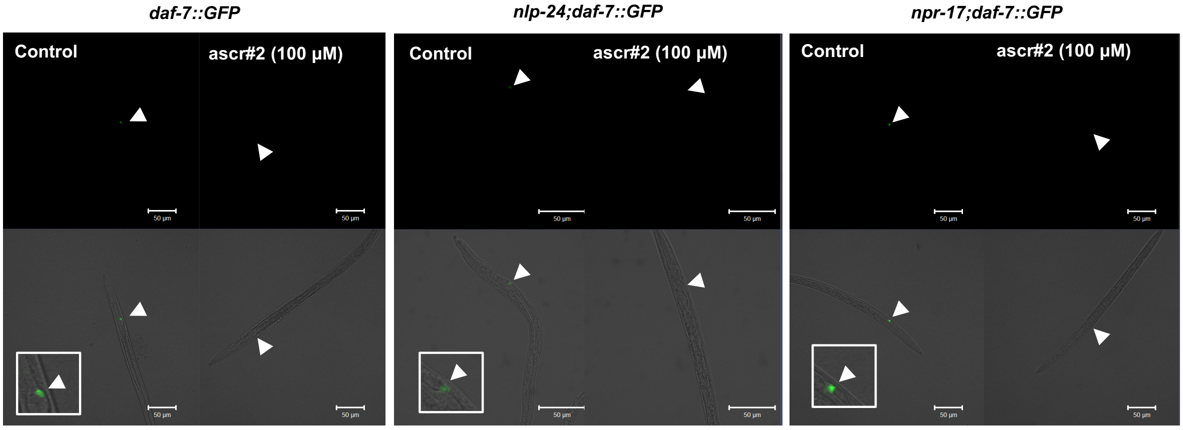

Supplementary Fig. S9. Expression of *daf-7* in opioid signaling mutants. The expression of DAF-7::GFP in the ASI neurons in the wildtype, *nlp-24(tm2105),* and *npr-17(tm3210)* backgrounds under control (fed) and starvation with ascr#2 (100µM) conditions. The images were taken using a Zeiss LSM880 confocal microscope with a 40x water-immersion lens under each condition. A white arrow indicates the position of the ASI neurons. Inserted images in teach controlled condition were magnified image of ASI neuron. Bar 50 µm.

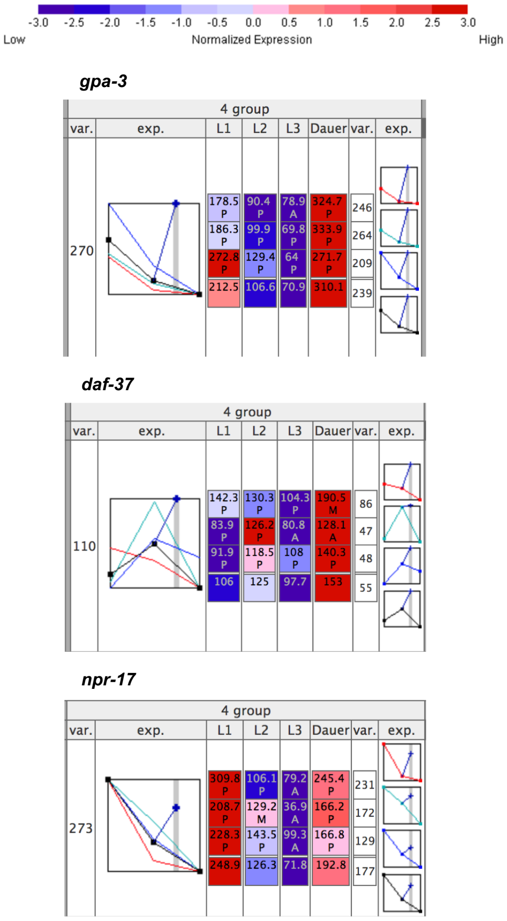

Supplementary Fig. S10. Growth stage-dependent expression patterns of *gpa-3*, *npr-17*, and *daf-37* from dauerDB. This Supplementary Figure shows that *gpa-3* and *daf-37*, essential components of dauer formation signaling, were highly increased in dauer larvae. On the other hand, the opioid receptor *npr-17* was not increased in dauer larvae (taken from www.dauerdb.org)[1].

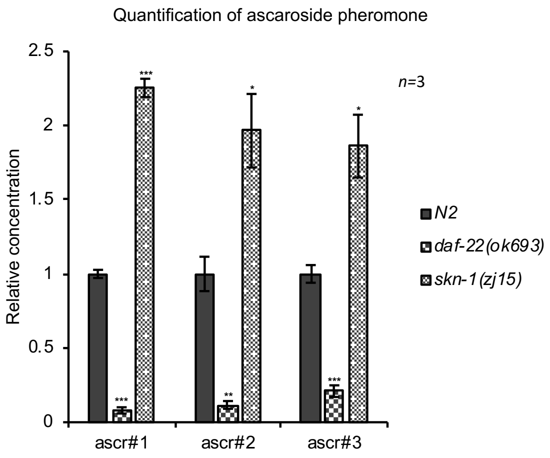

Supplementary Fig. S11. Pheromone biosynthesis in *skn-1(zj15)* worms. The amount of ascaroside pheromone biosynthesis in *skn-1(zj15)* worms was increased compared to that of wildtype worms (n=3). The data are represented as the mean with SEM of three different experiments. *, **, and *** indicate p-values < 0.05, < 0.01, and < 0.001, respectively, compared to N2. N.S. indicates no statistically significant difference. All p-values were calculated by unpaired t-test.

Supplementary Table S1. NLP-24::GFP expression in various condition and mutant background

Genetic Background/Mean±SEM of NLP-24::GFP intensity in ASI neuron (a.u.)	20℃					25℃				
	Fed	STS (3hr)		LTS (24hr)		Fed	STS (3hr)		LTS (24hr)	
	-	-	ascr#2 100μM	-	ascr#2 100μM	-	-	ascr#2 100μM	-	ascr#2 100μM
**WT *(Control line)***	53 ± 8	130 ± 11	87 ± 9	56 ± 11	9±1	134 ± 11	112 ± 11	116 ± 16	61 ± 15	21 ± 4
daf-22 (ok693)	16 ± 5	27 ± 6	40 ± 11	21 ± 6	18 ± 7	30 ± 3	45 ± 5	27 ± 4	22±4	14 ± 3
daf-22;Ex[ASK::daf-22]	139 ± 20	140 ± 18	95 ± 21	98 ± 13	61 ± 18	153 ± 18	199 ± 13	177 ± 16	152 ± 22	108 ± 22
npr-17(tm3210)	49 ± 8	61 ± 7	70 ± 9	53 ± 8	55 ± 10	138 ± 14	134 ± 13	115 ± 14	135 ± 21	139 ± 17
daf-37(ttTi3058)	18 ± 3	114 ± 12	126 ± 15	X	X	106 ± 16	98 ± 15	115 ± 17	X	X
daf-37;Ex[ASI::daf-37]	32 ± 8	32 ± 7	26 ± 6	X	X	60 ± 13	47 ± 10	32 ± 8	X	X
daf-37;Ex[ASK::daf-37]	38 ± 4	51 ± 7	33 ± 5	X	X	60 ± 3	57 ± 4	64 ± 4	X	X
gpa-3(pk35)	19 ± 4	20 ± 6	13 ± 2	X	X	47 ± 7	55 ± 9	44 ± 7	X	X
daf-16(mu86)	80 ± 13	98 ± 13	89 ± 15	55 ± 11	42 ± 9	145 ± 13	145 ± 15	109 ± 16	101 ± 15	124 ± 13
ser-1(ok345)	56 ± 10	27 ± 5	X	35 ± 7	X	19 ± 3	23 ± 3	X	21 ± 2	X
tph-1(mg280)	45 ± 10	56 ± 11	X	23 ± 4	X	90 ± 14	51 ± 13	X	79 ± 17	X
skn-1(zj15)	14 ± 3	24 ± 5	24 ± 5	96 ± 21	43 ± 12	54 ± 8	80 ± 12	108 ± 14	135 ± 22	200 ± 17
nhr-69(ok1926)	10 ± 2	21 ± 4	14 ± 3	90 ± 22	177 ± 20	58 ± 7	35 ± 8	39 ± 6	70 ± 20	112 ± 17

Supplementary Table S2. Prediction of GPCR-G protein coupling specificity (analyzed by PRED-COUPLE 2[4])

GPCR	Coupled G protein alpha subunit class	Normalized score*
DAF-37	$G_{i/o}$	0.96
	$G_{q/11}$	0.89
	$G_{12/13}$	0.05
	G_s	0.02
NPR-17	$G_{i/o}$	0.99
	$G_{q/11}$	0.02
	G_s	0.01
	$G_{12/13}$	0.00

* This value comes from the analysis of GPCR-binding probability using the PRED-COUPLE2 which is a method for the prediction of the coupling specificity of G-protein coupled receptors (GPCRs) to the four families of G-proteins[4]. According to the paper[4], this method is said to predict coupling to more than one family of G-proteins, as exemplified by several experimentally determined promiscuous receptors. It was described that the normalized score represents four numbers ranging from 0 to 1, which corresponds to the posterior probability that the GPCR under query couples to each of the four families of G-proteins. (Taken from http://athina.biol.uoa.gr/bioinformatics/PRED-COUPLE2/help.htm) [4].

Supplementary Table S3. Primers for genotyping.

Allele	Description	Sequence
nlp-24(tm2105)	tm2105 f<br>tm2105 r	GGTACGTCACGCTCGTTCAT<br>TGACCTTCCCAGTGTGGAGA
npr-17(tm3210)	tm3210 f<br>tm3210 r	GTGCAGGTTCAAATCCGTAG<br>GAGTCGATATCCACTGTGCA
daf-22(ok693)	ok693 f<br>ok693 r	TAAAAAAAAATTTTAAGAAATTTTTTAAATTGAAAA<br>GAGGTTGGACTTACCGGAAT
daf-22;psrbc-64::daf-22 cDNA	daf-22cDNA f<br>daf-22cDNA r	AAAGCGCAATTTTTAGAGCACAAA<br>AAGAAAATGTTTAAATTATTCCGTTTTTAT
daf-37(ttTi3058)	ttTi3058 f<br>ttTi3058 r	AGTTCTTTTCTGAAAATGAGAAAAAAG<br>CTGTCGTCTTTTACTCTTCGC
gpa-3(pk35)	pk35 f<br>pk35 r	GCAGAACCATATGTCACAGC<br>GATCTGTATCTGTTGCACACG
daf-2(e1370)	e1370 f<br>e1370 r	GAATCGTCAAGGATCATTTGATTATCGG<br>CGGAATGGCTCGTGATCTATTCTATCAT
daf-16(mu86)	mu86 f<br>mu86 m<br>mu86 r	ATGGCCTCAAGCATCACGTC<br>GGTCCAATGCCGGCAAAAA<br>GGAGCAATTGGTTCCGTCTG
ser-1(ok345)	ok345 f<br>ok345 r	CATAGCGAGTGTTTGGAGCA<br>AAGCATCTTTGAGCGCATTT
tph-1(mg280)	mg280 f<br>mg280 r	ATTCTTCTTCAATAAATTCGAAATCTGA<br>AGCGTCATCAGACGAGAGAC
skn-1(zj15)	zj15 f<br>zj15 r	CCACTCACTGCCGAAGAG<br>CATCGTTTGGTACAACTTCTGT
daf-7(e1372)	e1372 f<br>e1372 r	AACGAAATTCTCGACCAGCTG<br>TTGTTTGCTCGTCATACCTTTTG
nhr-69(ok1926)	ok1926 f<br>ok1926 r	ACACCCATCATACGCTCTCC<br>TGCTTTAACGGACGAGTTCA

Supplementary References

1. Jeong, P.-Y., Kwon, M.-S., Joo, H.-J. & Paik, Y.-K. Molecular Time-Course and the Metabolic Basis of Entry into Dauer in Caenorhabditis elegans. *PLOS ONE* **4**, e4162 (2009).
2. Niu, W. *et al.* Diverse transcription factor binding features revealed by genome-wide ChIP-seq in C. elegans. *Genome Res.* **21**, 245–254 (2011).
3. Van Nostrand, E. L. & Kim, S. K. Integrative analysis of C. elegans modENCODE ChIP-seq data sets to infer gene regulatory interactions. *Genome Res.* **23**, 941–953 (2013).
4. Sgourakis, N. G., Bagos, P. G., Papasaikas, P. K. & Hamodrakas, S. J. A method for the prediction of GPCRs coupling specificity to G-proteins using refined profile Hidden Markov Models. *BMC Bioinformatics* **6**, 104 (2005).
